# Low CD3+ and CD4+ T cell levels predict need for ventilatory support and in-hospital mortality in patients with COVID-19: a retrospective cohort study

**DOI:** 10.3389/fmed.2026.1740358

**Published:** 2026-01-26

**Authors:** Ester Lobato Martínez, Óscar Moreno-Pérez, Silvia Otero-Rodríguez, Raquel García-Sevila, Francisco Marco-de-la-Calle, Rosario Sánchez-Martínez, Esperanza Merino-de-Lucas, José-Manuel Ramos-Rincón

**Affiliations:** 1 Department of Internal Medicine, Alicante Institute for Health and Biomedical Research (ISABIAL), Dr. Balmis General University Hospital, Alicante, Spain; 2 Department of Endocrinology and Nutrition, Alicante Institute for Health and Biomedical Research (ISABIAL), Dr. Balmis General University Hospital, Alicante, Spain; 3 Department of Clinical Medicine, Miguel Hernández University, Alicante, Spain; 4 Infectious Diseases Unit, Alicante Institute for Health and Biomedical Research (ISABIAL), Dr. Balmis General University Hospital, Alicante, Spain; 5 Department of Pneumology, Alicante Institute for Health and Biomedical Research (ISABIAL), Dr. Balmis General University Hospital, Alicante, Spain; 6 Department of Immunology, Alicante Institute for Health and Biomedical Research (ISABIAL), Dr. Balmis General University Hospital, Alicante, Spain

**Keywords:** COVID-19, critical care, hospital mortality, interleukin 6, lymphocyte subsets, SARS-CoV-2, ventilatory support

## Abstract

**Objectives:**

The aim of the following study is to determine the association between lymphocyte subsets (total lymphocytes, CD3, CD4, CD8, B cells, NK cells) and clinical outcomes (need for non-invasive ventilatory support, ICU admission and in-hospital death) in patients hospitalized with SARS-CoV-2 infection.

**Methods:**

We conducted a single-center, pre-vaccination, retrospective cohort study including adults hospitalized between March 2020 and April 2021. Peripheral blood samples were collected within the first 24 h of admission for immune phenotyping. Additional clinical data were obtained from electronic health records. Statistical analyses included chi-square tests and multivariable logistic regression, adjusted for clinical characteristics and inflammatory biomarkers. Optimal cutoff points for immune and inflammatory markers were determined using the Youden index.

**Results:**

Among 959 patients, 29.4% required ventilatory support, 11.3% required ICU admission, and 10.7% died. In multivariable analysis adjusted by clinical and laboratory confounders, CD3+ cells (cutoff point: 666 cells/mm^3^) were independently associated with ventilatory support (aOR: 2.3, 95%CI: 1.5–3.4, *p* = 0.013) and in-hospital death (aOR: 2.4, 95%CI: 1.3–4.3, *p* = 0.048); and CD4+ cells (cutoff point: 359 cells/mm^3^) were independently associated with in-hospital death (aOR: 2.8, 95%CI: 1.4–5.5, *p* = 0.045).

**Conclusion:**

Adaptive immunity, especially T CD3+ and T CD4+ cells, is relevant in the prognosis of COVID-19, and T-cell counts can help identify hospitalized COVID-19 patients at risk for severe outcomes: ventilatory support and in-hospital death.

## 1 Introduction

Ever since the start of the pandemic in December 2019, the Coronavirus Disease 19 (COVID-19), caused by the severe acute respiratory syndrome coronavirus 2 (SARS-CoV-2), has caused more than 778 million cases and 7 million deaths worldwide (1). Although incidence and fatality rates have lowered with vaccination, the impact of COVID-19 is still relevant, with still increasing new cases and deaths as of September 2025 (1). In Spain, from March 2020 to June 2023, there were 681,927 hospital admissions due to COVID-19, with 56,249 intensive care unit (ICU) admissions and 121,852 deaths (2). Although the main target of SARS-CoV-2 is the respiratory system, COVID-19 is considered a systemic disease, with cardiovascular, renal, digestive and neurologic involvement (3). Furthermore, COVID-19 physiopathology involves not only direct viral damage, but also immune deregulation, triggering a proinflammatory cytokine response that can cause the phenomenon known as “cytokine storm,” which causes respiratory distress and multi organ failure (4).

In patients with severe SARS-CoV-2 infection, there is a delay of innate immune response, which fails to stimulate the adaptive immunity, represented by T helper (CD4+) and T cytotoxic (CD8+) cells (5). The impaired adaptive immunity, in turn, leaves a gap in the immune response to the infection that innate immunity tries to fill, which could lead to the “cytokine storm” (5). For that reason, several studies have focused on the role of lymphocyte subsets (TCD3+, TCD4+, TCD8+, B and NK cells) in COVID-19. The findings of these studies suggest that hospitalized patients with severe SARS-CoV-2 infection have lymphopenia or a high neutrophil/lymphocyte ratio (6). Furthermore, low levels of T cells, TCD4+ and TCD8+ subpopulations are associated with poor prognosis in COVID-19 (7, 8), with higher risk of mechanical ventilatory support and death (9). Some studies have also combined the lymphocyte subset counts with clinical data, like age or oxygen saturation (7) or other biomarkers, such as ferritin (7) or C reactive protein (10) to better predict severity and mortality in COVID-19.

However, these studies have some limitations that difficult the clinical application of the results. On the one hand, some studies are made with modest sample sizes (8–10), which influences effect size and statistical precision. On the other hand, lymphocyte levels are also influenced by clinical characteristics also associated with poor COVID-19 prognosis, such as age (7). Lastly, some studies do not use a cutoff point for lymphocyte counts (7, 8) or use different cutoffs depending on the method (9). Due to these limitations, although lymphocyte subsets have been studied in the literature, there is little information about the ideal cutoff to identify patients at risk of poor outcomes, and the influence of other clinical factors, like pharmacological treatments, is not clearly established.

The aim of this study is to assess the relationship between levels of lymphocyte subsets (CD3+, CD4+, CD8+, B, and NK cells) and severity of SARS-CoV-2 infection (ventilatory support. ICU admission and in-hospital death) in hospitalized patients with COVID-19 in a Spanish tertiary hospital, as well as to determine an optimal cutoff to stratify risk of poor outcomes in these patients.

## 2 Materials and methods

### 2.1 Study design, patients, and setting

This retrospective cohort study took place from March 2020 to April 2021 at the Dr. Balmis General University Hospital (Alicante, Spain), located on the southern Mediterranean coast. Eligible patients were hospitalized adults with confirmed SARS-CoV-2 infection using the reverse transcriptase polymerase chain reaction (RT-PCR) test, who gave their informed consent to participate in the study and had a good quality blood sample in the first 24 h of admission to perform the immune phenotyping. All consecutive patients enrolled in the study and with a viable blood sample for analysis were included.

### 2.2 Variables

Electronic medical records were reviewed to collect clinical variables, laboratory values in the initial routine blood analysis in the first 24 h of admission and patient outcomes.

#### 2.2.1 Clinical variables

The following clinical variables were collected: sex (male or female) and age on admission (in years). The Charlson comorbidity index (CCI) (11) was calculated, and for statistical analysis, patients were categorized as having no significant comorbidity (CCI 0–2 points) or significant comorbidity (CCI ≥ 3). Hypertension was defined as a history of pharmacologically treated hypertension. Diabetes referred to a history of type 1 or type 2 diabetes mellitus under pharmacological treatment. For hypertension and diabetes, the criteria included pharmacological treatment as they are objective data included in the electronic clinical records that allow to confirm and record the clinical variable in the database. Smoking history was classified as active, former, or no smoking history. Lung disease included a history of asthma, chronic obstructive pulmonary disease, pulmonary hypertension, sleep apnea–hypopnea syndrome, obesity hypoventilation syndrome, idiopathic pulmonary fibrosis, or other chronic lung diseases. Immunocompromise was defined as a history of primary or secondary immune deficiency; HIV infection with undetectable viral load and active treatment on admission was not considered immunocompromise. Oxygen saturation on room air on admission (SO_2_) was recorded and categorized as ≤ 94% or > 94% for analysis. The presence or absence of pneumonia at admission was defined by radiological findings on the initial chest X-ray or computer tomography (CT). Finally, pharmacological treatments received during hospitalization were recorded, including antibiotics (at least three doses), dexamethasone, tocilizumab, and remdesivir.

### 2.3 Laboratory values

The following laboratory parameters were recorded: interleukin 6 (IL-6, pg/mL), C-reactive protein (CRP, mg/dL), brain natriuretic peptide (proBNP, pg/mL), troponin T (TnT, ng/L), and ferritin (FT, mg/L).

#### 2.3.1 Outcomes

The following outcomes were assessed: (1) ventilatory support, defined as the need for non-invasive ventilatory support excluding low-flow nasal cannula and including bilevel positive airway pressure (BIPAP), continuous positive airway pressure (CPAP), and high-flow oxygen therapy through nasal cannula; (2) ICU admission, defined as any stay in a critical care unit during hospitalization; and (3) in-hospital death, defined as death occurring during hospitalization.

### 2.4 Immune phenotyping

Immune phenotyping analysis was performed by the Immunology Department of Dr. Balmis General University Hospital using standardized diagnostic four-color flow cytometry. For each participant, two 50 μl peripheral blood samples preserved in ethylenediamine tetraacetic acid (EDTA) were analyzed. One for the determination of T lymphocyte subpopulations (CD3+CD4+ and CD3+CD8+), and the other for total T cells (CD3+), B cells (CD19+), and NK cells (CD16+ CD56+).

On the one hand, for the determination of CD3+CD4+ and CD3+CD8+ subsets, standardized diagnostic four-color flow cytometry was used with a commercial *in vitro* diagnostic (IVD) lyse–non-wash method (Multitest, BD Biosciences™, San Jose, CA, United States) and a FACS Canto II flow cytometer (BD Biosciences). The Multitest kit contains the following conjugated monoclonal antibodies: CD3-FITC clone SK7, CD8-PE clone SK1, CD45-PerCP clone 2D1, and CD4-APC clone SK3.

On the other hand, for the determination of CD3+, CD19+ and CD16+ CD56+ cells, a lyse-wash method was used, with the following IVD-grade conjugated antibodies: CD45-PerCP clone 2D1 (BD Biosciences), CD3-APC clone SK7 (BD Biosciences), CD19-FITC clone SJ25C1 (BD Biosciences), CD56-PE clone C5.9 (Cytognos SL, Salamanca, Spain), and CD16-PE clone 3G8 (BD Biosciences).

The laboratory procedures, mixing, incubating and lyse-wash methods were performed according to the manufacturer’s instructions. In both cases, samples were analyzed on a FACS Canto II flow cytometer (BD Biosciences) using FACS Canto™ Clinical Software (BD Biosciences), which allows for the automatic identification of lymphocytes in the sample and the determination and gating of the different subsets using successive dot plots. In each assay, a minimum of 2,500 lymphocytes were analyzed. The gating strategy and hierarchy for each lymphocyte subset is detailed in Supplementary Figure 1.

### 2.5 Statistical analysis

#### 2.5.1 Descriptive analysis

For the descriptive analysis, categorical variables were expressed as frequencies (percentages) and continuous variables as medians (interquartile range, IQR).

#### 2.5.2 Univariable analysis

Categorical variables were analyzed using the chi square test, measuring the association with odds ratio (OR) and 95% confidence interval (95%CI). For the statistical analysis, age was coded into a dichotomous variable: 18–64 and 65 or higher. The analysis of quantitative variables (laboratory markers and lymphocyte subsets) comprised the following steps:

(1)    Univariable analysis as a quantitative variable using the Mann-Whitney U test, for each clinical outcome (ventilatory support, ICU admission and in-hospital death) as dependent variables.
(2)    For every quantitative variable and statistically significant outcome, a ROC curve was calculated, to determine the area under the curve (AUC) and the cutoff point with higher Youden index (sensitivity + specificity – 1). Each quantitative variable could yield up to three different cutoff points, one for each outcome.
(3)    Determination of a final cutoff value for every quantitative variable from the obtained cutoff points in the former step, using the mean, if the coefficient of variation (CV) was lower than 30%, or the median, if the CV was 30% or higher.
(4)    Categorization of the laboratory markers and lymphocyte subsets using the final cutoff value.
(5)    Univariable analysis of laboratory markers and lymphocyte subsets as a categorical value stratified by the final cutoff value with chi-square test, reporting association as OR (95%CI).

#### 2.5.3 Multivariable analysis

A binary logistic regression model was built for every outcome as dependent variable (ventilatory support, ICU admission and in-hospital death), including the following covariables:

(1)    Sex, age (as a dichotomous variable, 18–64 and 65 or older) and CCI (as a dichotomous variable, 0–2 and 3 or more), in all models;
(2)    Clinical variables with a statistically significant result (OR and 95% CI) in the univariable analysis: oxygen saturation and radiological findings;
(3)    Laboratory markers with a statistically significant association (OR and 95% CI) in the univariable analysis, entered as categorical variables using the final cutoff. If interleukin 6 was included in the model, use of tocilizumab was added to avoid potential confounding;
(4)    Total lymphocytes and lymphocyte subsets with a statistically significant association in the univariable analysis (OR and 95% CI), entered as categorical variables using the final cutoff. Because total lymphocytes and CD3+ include other lymphocyte subpopulations, they were analyzed separately to avoid collinearity; therefore, for every outcome, three different models were built:

Model 1: CD4+ cells, CD8+ cells, B cells, NK cells, plus sex, age, CCI, clinical variables and laboratory markers.

Model 2: Total lymphocytes plus sex. age, CCI, clinical variables and laboratory markers.

Model 3: CD3+ cells plus sex. age, CCI, clinical variables and laboratory markers.

To control type I error due to multiple comparisons, Bonferroni correction was applied within each family of hypotheses using the following formula:


$$p^{*}=\min\left(1,p\times m\right)$$

Where p* is the adjusted *p*-value, p is the original *p*-value and m is the number of simultaneous hypotheses tests within each family (number of group/effect comparisons x number of outcomes). Results were considered statistically significant if p* < 0.05.

All statistical analyses were performed using IBM SPSS Statistics for Windows, Version 26.0 (Armonk, NY, United States, IBM Corp.).

### 2.6 Ethical aspects

This work was approved by the institutional research ethics committee of Dr. Balmis General University Hospital, (reference number 200145). All patients gave their informed consent before participating in the study. The research was conducted according to the principles of the Declaration of Helsinki.

## 3 Results

### 3.1 Baseline characteristics

Of the 1,182 patients who consented to participate in the study, 959 had a viable blood sample stored in the first 24 h of admission and therefore constitute the cohort of this study. Baseline characteristics of the study cohort, which are representative of the baseline population, are summarized in Table 1. The cohort is comprised of 43% of women, a median age of 67 years and more than half have significant comorbidity, defined by at least 3 points in the CCI. Oxygen saturation on room air was lower than 94% in 42.4% of patients and 82.1% had pneumonia on the initial X-ray. During their hospital stay, 29.3% of patients required ventilatory support, 11.3% were admitted in the ICU, and 10.7% died. Almost 90% of the patients received dexamethasone, 75% at least 3 doses of antibiotic and 30% tocilizumab.

**TABLE 1:** Baseline characteristics of the study cohort.

Clinical characteristics, n/N (%)		Baseline population	Study cohort
Sex	Female	527/1,182 (44.6)	420/959 (43.8)
	Male	655/1,182 (55.4)	539/959 (56.2)
Age	Median (range)	67 (18–100)	67 (18–100)
	18–64	528/1,182 (44.7)	439/959 (45.8)
	≥ 65	654/1,182 (55.3)	520/959 (54.2)
Charlson comorbidity index	0–2	490/1,174 (41.7)	405/951 (42.6)
	≥ 3	684/1,174 (58.3)	546/951 (57.4)
Hypertension	Yes	618/1,180 (52.4)	505/957 (52.8)
	No	562/1,180 (47.6)	452/957 (47.2)
Diabetes	Yes	322/1,180 (27.3)	257/957 (26.9)
	No	858/1,180 (72.7)	700/957 (73.1)
Smoking history	Yes	118/1,136 (10.4)	96/931 (10.3)
	No	1,018/1,136 (89.6)	835/931 (89.7)
Lung disease	Yes	205/1,177 (17.4)	171/955 (17.9)
	No	972/1,177 (82.6)	784/955 (82.1)
Immunocompromise	Yes	84/1,181 (7.1)	68/958 (7.1)
	No	1,097/1,181 (92.9)	890/958 (92.9)
SO_2_	≤ 94%	503/1,110 (45.3)	382/902 (42.4)
	> 94%	607/1,110 (54.7)	520/902 (57.6)
Radiological findings	Normal	212/1,157 (18.3)	168/938 (17.9)
	Pneumonia	945/1,157 (81.7)	770/938 (82.1)
**Outcomes, n/N (%)**		**Baseline population**	**Study cohort**
Ventilatory support	Yes	363/1,164 (31.2)	277/946 (29.3)
	No	801/1,164 (68.8)	669/946 (70.7)
ICU admission	Yes	167/1,181 (14.1)	108/959 (11.3)
	No	1014/1,181 (85.9)	851/959 (88.7)
In-hospital death	Yes	149/1,176 (12.7)	102/955 (10.7)
	No	1027/1,176 (87.3)	853/955 (89.3)
Treatment, n/N (%)		**Baseline population**	**Study cohort**
Antibiotic	Yes	851/1,134 (75)	705/931 (75.7)
	No	283/1,134 (25)	226/931 (24.3)
Dexamethasone	Yes	1,011/1,135 (89.1)	837/931 (89.9)
	No	124/1,135 (10.9)	94/931 (10.1)
Tocilizumab	Yes	370/1,173 (31.5)	309/953 (32.4)
	No	803/1,173 (68.5)	644/953 (67.6)
Remdesivir	Yes	164/1,134 (14.5)	141/930 (15.2)
	No	970/1,134 (85.5)	789/930 (84.8)
**Laboratory values, median (range), study cohort**			
Total lymphocytes, cells/mm^3^	980 (66–11,460)		
T CD3+ cells, cells/mm^3^	673 (66–3,998)		
T CD4+ cells, cells/mm^3^	415.5 (38–2,018)		
T CD8+ cells, cells/mm^3^	224 (15–3,035)		
B cells, cells/mm^3^	98 (0–86,955)		
NK cells, cells/mm^3^	169 (2–2,525)		
IL-6, pg/mL	27 (2–2,995)		
CRP, mg/dL	5.9 (0.1–118)		
proBNP, pg/mL	156 (0–35,000)		
TnT, ng/L	10 (0–4,061)		
DD, mg/mL	0.83 (0.08–77.698)		
FT, mg/L	669 (2–10,478)		

SO_2_, oxygen saturation; n/N, proportion; %, percentage; IL-6, interleukin 6; CRP, C reactive protein; proBNP, brain natriuretic peptide; TnT, troponin T; DD, D dimer; FT, ferritin.

### 3.2 Univariable analysis of categorical variables

Univariable analysis of categorical variables, with OR and 95%CI for all three outcomes is shown in Table 2. The clinical variables associated with ventilatory support are male sex (OR 1.37, 95%CI 1.11–1.68, *p* = 0.003), age 65 years or higher (OR 1.85, 95%CI 1.48–2.29, *p* < 0.001), CCI 3 points or higher (OR 1.87, 95%CI 1.49–2.35, *p* < 0.001), hypertension (OR 1.36, 95%CI 1.11–1.67, *p* = 0.003), diabetes (OR 1.57, 95%CI 1.29–1.91, *p* < 0.001), low oxygen saturation (OR 2.6, 95%CI 2.08–3.26, *p* < 0.001), lung disease (OR 1.51, 95%CI 1.21–1.87, *p* < 0.001) and presence of pneumonia on admission (OR 1.46, 95%CI 1.07–1.99, *p* = 0.013). The only clinical variable associated with higher ICU admission is low oxygen saturation (OR 2.24, 95%CI 1.52–3.3, *p* < 0.001). Risk factors for in-hospital death are age 65 years or higher (OR, 95%CI, *p* < 0.001), CCI 3 points or higher (OR 9.9, 95%CI 4.63–21.28, *p* < 0.001), hypertension (OR 1.96, 95%CI 1.31–2.91, *p* < 0.001), diabetes (OR 2.24, 95%CI 2.24, *p* < 0.001), low oxygen saturation (OR 2.25, 95%CI 1.51–3.36, *p* < 0.001), lung disease (OR 1.64, 95%CI 1.1–2.47, *p* = 0.02) and immunocompromise (OR 2.07, 95%CI 1.25–3.44, *p* = 0.006).

**TABLE 2:** Univariate analysis of categorical variables for ventilatory support, ICU admission and in-hospital death.

Ventilatory support		n/N (%)	OR (95%CI)	*P*
Sex	Female	100/412 (24.3)	1	0.003*
	Male	177/534 (33.1)	1.37 (1.11–1.68)	
Age	18–64	87/433 (20.1)	1	<0.001*
	≥ 65	190/513 (37)	1.85 (1.48–2.29)	
Charlson comorbidity index	0–2	78/401 (19.5)	1	<0.001*
	≥ 3	195/537 (36.3)	1.87 (1.49–2.35)	
Hypertension	No	109/445 (24.5)	1	0.003*
	Yes	166/499 (33.3)	1.36 (1.11–1.67)	
Diabetes	No	174/689 (25.3)	1	<0.001*
	Yes	101/255 (39.6)	1.57 (1.29–1.91)	
Smoking history	No	254/824 (30.8)	1	0.06
	Yes	20/94 (21.3)	0.69 (0.46–1.03)	
Lung disease	No	208/776 (26.8)	1	<0.001*
	Yes	67/166 (40.4)	1.51 (1.21–1.87)	
Immunocompromise	No	256/877 (29.2)	1	0.99
	Yes	20/68 (29.4)	1.01 (0.69–1.48)	
SO_2_	> 94%	86/512 (16.8)	1	<0.001*
	≤ 94%	166/380 (43.7)	2.6 (2.08–3.26)	
Radiological findings	Normal	35/164 (21.3)	1	0.013*
	Pneumonia	238/765 (31.1)	1.46 (1.07–1.99)	
Antibiotic	No	36/224 (16.1)	1	<0.001*
	Yes	240/697 (34.4)	2.14 (1.56–2.94)	
Dexamethasone	No	9/92 (9.8)	1	<0.001*
	Yes	268/829 (32.3)	3.3 (1.76–6.19)	
Tocilizumab	No	77/637 (12.1)	1	<0.001*
	Yes	200/308 (64.9)	5.38 (4.29–6.71)	
Remdesivir	No	215/780 (27.6)	1	<0.001*
	Yes	61/140 (43.6)	1.58 (1.27–1.97)	
ICU admission		**n/N (%)**	**OR (95%CI%)**	**p**
Sex	Female	42/420 (10)	1	0.28
	Male	66/539 (12.2)	1.05 (0.74–1.51)	
Age	18–64	48/439 (10.9)	1	0.77
	≥ 65	60/520 (11.5)	2.10 (1.38–3.20)	
Charlson comorbidity index	0–2	50/405 (12.3)	1	0.36
	≥ 3	57/546 (10.4)	0.98 (0.93–1.03)	
Hypertension	No	47/452 (10.4)	1	0.41
	Yes	61/505 (12.1)	1.16 (0.81–1.66)	
Diabetes	No	71/700 (10.1)	1	0.07
	Yes	37/257 (14.4)	1.42 (0.98–2.06)	
Smoking history	No	93/835 (11.1)	1	0.32
	Yes	14/96 (14.6)	1.31 (0.78–2.2)	
Lung disease	No	84/784 (10.7)	1	0.30
	Yes	23/171 (13.5)	1.26 (0.82–1.93)	
Immunocompromise	No	102/890(11.5)	1	0.51
	Yes	6/68 (8.8)	0.77 (0.35–1.69)	
SO_2_	> 94%	37/520 (7.1)	1	<0.001*
	≤ 94%	61/382 (16)	2.24 (1.52–3.3)	
Radiological findings	Normal	13/168 (7.7)	1	0.11
	Pneumonia	93/770 (12.1)	1.56 (0.9–2.72)	
Antibiotic	No	7/226 (3.1)	1	<0.001*
	Yes	101/705 (14.3)	4.62 (2.18–9.80)	
Dexamethasone	No	5/94 (5.3)	1	0.05
	Yes	103/837 (12.3)	2.31 (0.97–5.53)	
Tocilizumab	No	26/644 (4)	1	<0.001*
	Yes	82/309 (26.5)	6.58 (4.32–10)	
Remdesivir	No	93/789 (11.8)	1	0.70
	Yes	15/141 (10.6)	0.9 (0.54–1.51)	
In-hospital death		**n/N (%)**	**OR (95%CI)**	**p**
Sex	Female	42/418 (10)	1	0.58
	Male	60/537 (11.2)	1.11 (0.77–1.61)	
Age	18–64	7/438 (1.6)	1	<0.001*
	≥ 65	95/517 (18.4)	11.49 (5.41–24.39)	
Charlson comorbidity index	0–2	7/404 (1.7)	1	<0.001*
	≥ 3	93/543 (17.1)	9.9 (4.63–21.28)	
Hypertension	No	32/450 (7.1)	1	<0.001*
	Yes	70/503 (13.9)	1.96 (1.31–2.91)	
Diabetes	No	56/697 (8)	1	<0.001*
	Yes	46/256 (18)	2.24 (1.56–3.21)	
Smoking history	No	90/831 (10.8)	1	0.85
	Yes	11/96 (11.5)	1.06 (0.59–1.91)	
Lung disease	No	75/780 (9.6)	1	0.02*
	Yes	27/171 (15.8)	1.64 (1.1–2.47)	
Immunocompromise	No	88/886 (9.9)	1	0.006*
	Yes	14/68 (20.6)	2.07 (1.25–3.44)	
SO_2_	> 94%	35/518 (6.8)	1	<0.001*
	≤ 94%	58/381 (15.2)	2.25 (1.51–3.36)	
Radiological findings	Normal	25/168 (14.9)	1	0.039*
	Pneumonia	73/768 (9.5)	0.68 (0.42–0.97)	
Antibiotic	No	13/226 (5.8)	1	0.004*
	Yes	89/703 (12.7)	2.2 (1.25–3.86)	
Dexamethasone	No	8/94 (8.5)	1	0.44
	Yes	93/835 (11.1)	1.31 (0.66–2.61)	
Tocilizumab	No	48/643 (7.5)	1	<0.001*
	Yes	54/308 (17.5)	2.35 (1.63–3.38)	
Remdesivir	No	87/787 (11.1)	1	0.88
	Yes	15/141 (10.6)	0.96 (0.57–1.62)	

n/N, proportion; OR, odds ratio; 95%CI, 95% confidence interval; p, *p*-value; SO_2_, oxygen saturation; p, *p*-value. Significant *p*-values are highlighted in bold and marked with an asterisk (*).

The presence of pneumonia on admission is associated to lower risk of death in the univariable analysis (OR 0.68, 95%CI 0.42–0.97, *p* = 0.039). Likewise, the treatment variables are associated in the univariable analysis to poor outcomes, without considering the interaction with other variables: antibiotic (OR 2.14, 95%CI 1.56–2.94, *p* < 0.001), dexamethasone (OR 3.3, 95%CI 1.76–6.19, *p* < 0.001), tocilizumab (OR 5.38, 95%CI 4.29–6.71, *p* < 0.001) and remdesivir (OR 1.58, 95%CI 1.27–1.97, *p* < 0.001) for ventilatory support; antibiotic (OR 4.62, 95%CI 2.18–9.80, *p* < 0.001) and tocilizumab (OR 6.58, 95%CI 4.32–10, *p* < 0.001) for ICU admission; and antibiotic (OR 2.2, 95%CI 1.25–3.86, *p* = 0.004) and tocilizumab (OR 2.35, 95%CI 1.63–3.38, *p* < 0.001) for in-hospital death.

### 3.3 Univariable analysis of quantitative variables

Univariable analysis of laboratory values and lymphocyte populations with a non-parametric quantitative test can be found in Table 3. Patients with poor outcomes have lower median levels of total lymphocytes (ventilatory support: 870 vs. 1,030 cells/mm^3^, *p* < 0.001; ICU admission: 855 vs. 1,005 cells/mm^3^, *p* < 0.001; in-hospital death: 770 vs. 1,010 cells/mm^3^, *p* < 0.001), CD3+ cells (ventilatory support: 549 vs. 750 cells/mm^3^, *p* < 0.001; ICU admission: 561.5 vs. 697.5 cells/mm^3^, *p* < 0.001; in-hospital death: 438 vs. 707 cells/mm^3^, *p* < 0.001), CD4+ cells (ventilatory support: 330 vs. 464 cells/mm^3^, *p* < 0.001; ICU admission: 342 vs. 439 cells/mm^3^, *p* < 0.001; in-hospital death: 250 vs. 452 cells/mm^3^, *p* < 0.001) and CD8+ cells (ventilatory support: 192 vs. 252 cells/mm^3^, *p* < 0.001; ICU admission: 188.5 vs. 237 cells/mm^3^, *p* < 0.001; in-hospital death: 167 vs. 237 cells/mm^3^, *p* < 0.001).

**TABLE 3:** Univariable analysis of laboratory values and lymphocyte populations (quantitative test).

Variable	Ventilatory support			ICU admission			In-hospital death		
	Yes, median (p75–p25)	No, median (p75–p25)	*p*	Yes, median (p75–p25)	No, median (p75–p25)	*p*	Yes, median (p75–p25)	No, median (p75–p25)	*p*
Total lymphocytes	870 (610)	1,030 (640)	< 0.001*	855 (505)	1,005 (660)	< 0.001*	770 (540)	1,010 (630)	< 0.001*
CD3+	549 (423)	750 (565)	< 0.001*	561.5 (414)	697.5 (565)	< 0.001*	438 (402)	707 (548)	< 0.001*
CD4+	330 (298)	464 (404)	< 0.001*	342 (308)	439 (379)	< 0.001*	250 (255)	452 (367)	< 0.001*
CD8+	192 (174)	252 (230)	< 0.001*	188.5 (140)	237 (226)	< 0.001*	167 (178)	237 (218)	< 0.001*
B	73 (99)	105 (117)	< 0.001*	94.5 (110)	98 (110)	0.331	61 (79)	102 (118)	< 0.001*
NK	160 (168)	173 (137)	0.031*	143.5 (140)	173 (146)	0.006*	148 (176)	172 (144)	0.124
IL-6	59 (116)	20 (40)	< 0.001*	68.5 (152)	24 (51)	< 0.001*	55.5 (118)	24 (52)	< 0.001*
CRP	8.71 (10.5)	5.02 (7.2)	< 0.001*	9.805 (13.1)	5.69 (7.6)	< 0.001*	8.18 (10.6)	5.76 (7.6)	< 0.001*
proBNP	310 (924)	115 (429)	< 0.001*	130.5 (302)	156.5 (587)	0.139	758 (2,581)	128 (436)	< 0.001*
TnT	15 (22)	9 (14)	< 0.001*	10.5 (11)	10 (18)	0.947	29 (37.8)	9 (14)	< 0.001*
DD	0.94 (1.44)	0.78 (0.91)	< 0.001*	0.71 (0.62)	0.825 (1.05)	0.370	1.26 (2.15)	0.79 (0.93)	< 0.001*
FT	843 (1,141)	645 (873)	< 0.001*	1123.5 (1,286)	662 (909)	< 0.001*	769 (958)	681.5 (970)	0.441

p75, 75th percentile; p25, 25th percentile; p, *p*-value; IL-6, interleukin 6; CRP, C reactive protein; proBNP, brain natriuretic peptide; TnT, troponin T; DD, D dimer; FT, ferritin. Significant *p*-values are highlighted in bold and marked with an asterisk (*).

Median B cells are lower in all outcomes except in ICU admission (ventilatory support: 73 vs. 105 cells/mm^3^, *p* < 0.001; in-hospital death: 61 vs. 102 cells/mm^3^, *p* < 0.001), and median NK cells are lower in all outcomes except for in-hospital death (ventilatory support: 160 vs. 173 cells/mm^3^, *p* = 0.031; ICU admission: 143.5 vs. 173 cells/mm^3^, *p* = 0.006). Median IL-6 levels are higher in patients with poor outcomes (ventilatory support: 59 vs. 20 pg/mL, *p* < 0.001; ICU admission: 68.5 vs. 24 pg/mL, *p* < 0.001; in-hospital death: 55.5 vs. 24 pg/mL, *p* < 0.001).

Patients with need of ventilatory support have higher median levels of CRP (8.71 vs. 5.02 mg/dL, *p* < 0.001), proBNP (310 vs. 115 pg/mL, *p* < 0.001), TnT (15 vs. 9 ng/L, *p* < 0.001), DD (0.94 vs. 0.78 mg/mL, *p* < 0.001) and FT (843 vs. 645 mg/L, *p* < 0.001). Median CRP (9.81 vs. 5.69 mg/dL, *p* < 0.001) and FT (1,123.5 vs. 662 mg/L, *p* < 0.001) are higher in patients admitted in the ICU. Patients who died in hospitalization have higher median levels of CRP (8.18 vs. 5.76 mg/dL, *p* < 0.001), proBNP (758 vs. 128 pg/mL, *p* < 0.001), TnT (29 vs. 9 ng/L, *p* < 0.001) and DD (1.26 vs. 0.79 mg/mL, *p* < 0.001).

Table 4 shows the AUC, optimal cutoff, sensitivity and specificity for laboratory values and lymphocyte populations. The highest Youden index is of 0.35 for CD4+ cells and in-hospital death, with a cutoff value of 289.5 cells/mm^3^, sensitivity of 59.6% and specificity of 75%. Grouped cutoff points for every biomarker with coefficient of variation and designated common cutoff can be seen in Table 5. The defined cutoffs for stratified univariable and multivariable analysis are: 948 cells/mm^3^, for total lymphocytes; 666 cells/mm^3^, for CD3+ cells; 359 cells/mm^3^, for CD4+ cells; 241 cells/mm^3^, for CD8+ cells; 77 cells/mm^3^, for B cells; 117 cells/mm^3^, for NK cells; 39.5 pg/mL, for IL-6; 5.3 mg/dL, for CRP; 246 pg/mL, for proBNP; 12.5 ng/L, for TnT; 0.83 mg/mL, for DD; and 810 mg/L, for FT.

**TABLE 4:** Optimal cutoff points and AUC values for laboratory values and lymphocyte populations.

Variable	Ventilatory support					
	AUC (95%CI)	Optimal cutoff	S	Sp	Youden	Quality
Total lymphocytes	0.61 (0.57–0.65)	994.5	66.2%	54.7%	0.21	0.57
CD3+	0.67 (0.62–0.7)	673	69.3%	57.9%	0.27	0.62
CD4+	0.65 (0.61–0.69)	453	71.5%	52.2%	0.24	0.61
CD8+	0.63 (0.59–0.67)	269.5	74.4%	45.3%	0.2	0.59
B	0.61 (0.57–0.65)	77.5	53.6%	66.3%	0.2	0.56
NK	0.55 (0.5–0.59)	112.5	39.4%	75%	0.16	0.5
IL-6	0.71 (0.68–0.75)	51.5	56%	77%	0.33	0.68
CRP	0.65 (0.61–0.69)	5.2	72.6%	51.7%	0.24	0.61
proBNP	0.62 (0.58–0.66)	187	63.2%	60.2%	0.23	0.58
TnT	0.63 (0.6–0.68)	8.5	75.4%	48.9%	0.24	0.6
DD	0.58 (0.54–0.62)	0.83	60.9%	54.3%	0.15	0.54
FT	0.58 (0.54–0.62)	734.5	56.3%	58.3%	0.15	0.54
**Variable**	**ICU admission**					
	**AUC (95%CI)**	**Optimal cutoff**	**S**	**Sp**	**Youden**	**Quality**
Total lymphocytes	0.62 (0.56–0.67)	994.5	71%	51.1%	0.22	0.56
CD3+	0.62 (0.56–0.67)	781.5	76.6%	57.6%	0.34	0.56
CD4+	0.60 (0.55–0.66)	334.5	53.3%	64.7%	0.18	0.55
CD8+	0.61 (0.55–0.66)	244.5	72%	47.3%	0.19	0.55
NK	0.58 (0.52–0.65)	121.5	45.5%	70.5%	0.16	0.52
IL-6	0.71 (0.66–0.76)	33.5	72.2%	60.9%	0.33	0.66
CRP	0.64 (0.59–0.7)	13.4	39.6%	83.4%	0.23	0.59
FT	0.6 (0.55–0.66)	885.5	56.3%	64%	0.2	0.54
**Variable**	**In-hospital death**					
	**AUC (95%CI)**	**Optimal cutoff**	**S**	**Sp**	**Youden**	**Quality**
Total lymphocytes	0.65 (0.59–0.71)	855	61.4%	65.3%	0.27	0.59
CD3+	0.69 (0.63–0.75)	542.5	63.6%	69%	0.33	0.63
CD4+	0.71 (0.66–0.76)	289.5	59.6%	75%	0.35	0.66
CD8+	0.63 (0.57–0.7)	207.5	65.7%	56.4%	0.22	0.57
B	0.68 (0.62–0.73)	75.5	63.6%	64.5%	0.28	0.62
IL-6	0.7 (0.65–0.75)	33.5	71.6%	60.5%	0.32	0.65
CRP	0.61 (0.55–0.67)	5.3	71.3%	48.5%	0.2	0.55
proBNP	0.74 (0.69–0.8)	305.5	77.4%	67.5%	0.45	0.69
TnT	0.81 (0.77–0.85)	16.5	81.5%	71.1%	0.53	0.77
DD	0.65 (0.59–0.71)	0.84	74.2%	53.1%	0.27	0.59

AUC, area under the curve; S, sensitivity; Sp, specificity; IL-6, interleukin 6; CRP, C reactive protein; proBNP, brain natriuretic peptide; TnT, troponin T; DD, D dimer; FT, ferritin.

**TABLE 5:** Final cutoff values for laboratory values and lymphocyte populations.

Variable	Ventilatory support	ICU admission	In-hospital death	Mean	Std. Dev.	CV (%)	Final cutoff value
Total lymphocytes	994.5	994.5	855	948.00	65.76	6.94	**948**
CD3+	673	781.5	542.5	665.67	97.71	14.68	**666**
CD4+	453	334.5	289.5	359.00	68.96	19.21	**359**
CD8+	269.5	244.5	207.5	240.50	25.47	10.59	**241**
B	77.5	-	75.5	76.50	1.00	1.31	**77**
NK	112.5	121.5	-	117.00	4.50	3.85	**117**
IL-6	51.5	33.5	33.5	39.50	8.49	21.48	**39.5**
CRP	5.2	13.39	5.3	7.97	3.84	48.14	**5.3**
proBNP	187	-	305.5	246.25	59.25	24.06	**246**
TnT	8.5	-	16.5	12.50	4.00	32.00	**12.5**
DD	0.83	-	0.84	0.83	0.01	0.60	**0.83**
FT	734.5	885.5	-	810.00	75.50	9.32	**810**

Std. Dev., standard deviation; CV, coefficient of variation; IL-6, interleukin 6; CRP, C reactive protein; proBNP, brain natriuretic peptide; TnT, troponin T; DD, D dimer; FT, ferritin. Final cutoff values are highlighted in bold.

Univariable analysis of laboratory values and lymphocyte populations stratified by final cutoff values is summarized in Table 6. Patients with lymphocyte subsets below the established cutoff of all have higher risk of ventilatory support (total lymphocytes, OR 1.71, 95% CI: 1.39–2.1, *p* < 0.001; CD3+cells, OR 2.15, 95% CI: 1.72–2.67, *p* < 0.001; CD4+ cells, OR 1.86, 95% CI: 1.52–2.27, *p* < 0.001; CD8+ cells, OR 1.67, 95% CI: 1.34–2.08, *p* < 0.001; B cells, OR 1.8, 95% CI: 1.45–2.23, *p* < 0.001; NK cells, OR 1.53, 95% CI: 1.24–1.9, *p* < 0.001); ICU admission, except B cells (total lymphocytes, OR 1.84, 95% CI: 1.27–2.66, *p* < 0.001; CD3+cells, OR 1.89, 95% CI: 1.3–2.74, *p* < 0.001; CD4+ cells, OR 1.77, 95% CI: 1.24–2.54, *p* = 0.002; CD8+ cells, OR 1.99, 95% CI: 1.34–2.95, *p* < 0.001; B cells, OR 1.26, 95% CI: 0.87–1.83, *p* = 0.23; NK cells, OR 1.64, 95% CI: 1.13–2.38, *p* = 0.01); and in-hospital death (total lymphocytes, OR 2.37, 95% CI: 1.59–3.51, *p* < 0.001; CD3+cells, OR 2.92, 95% CI: 1.9–4.48, *p* < 0.001; CD4+ cells, OR 3.03, 95% CI: 2.03–4.52, *p* < 0.001; CD8+ cells, OR 1.96, 95% CI: 1.3–2.95, *p* < 0.001; B cells, OR 2.7, 95% CI: 1.79–4.07, *p* < 0.001; NK cells, OR 1.65, 95% CI: 1.11–2.46, *p* = 0.01).

**TABLE 6:** Univariable analysis of laboratory values and lymphocyte populations stratified by final cutoff values.

Ventilatory support		n/N (%)	OR (95%CI)	*p*
Total lymphocytes, cells/mm^3^	**≤ 948**	165/438 (37.7)	1.71 (1.39–2.1)	<0.001*
	**> 948**	110/499 (22)	1	
CD3+, cells/mm^3^	**≤ 666**	182/454 (40.1)	2.15 (1.72–2.67)	<0.001*
	**> 666**	88/471 (18.7)	1	
CD4+, cells/mm^3^	**≤ 359**	152/379 (40.1)	1.86 (1.52–2.27)	<0.001*
	**> 359**	118/546 (21.6)	1	
CD8+, cells/mm^3^	**≤ 241**	179/500 (35.8)	1.67 (1.34–2.08)	<0.001*
	**> 241**	91/425 (21.4)	1	
B, cells/mm^3^	**≤ 77**	126/333 (37.8)	1.8 (1.45–2.23)	<0.001*
	**> 77**	109/517 (21.1)	1	
NK, cells/mm^3^	**≤ 117**	95/260 (36.5)	1.53 (1.24–1.9)	<0.001*
	**> 117**	141/592 (23.8)	1	
IL-6, pg/mL	**≤ 39.5**	110/585 (18.8)	1	<0.001*
	**> 39.5**	167/361 (46.3)	2.46 (2.01–3.01)	
CRP, mg/dL	**≤ 5.3**	83/432 (19.2)	1	<0.001*
	**> 5.3**	194/512 (37.9)	1.97 (1.58–2.46)	
proBNP, pg/mL	**≤ 246**	116/525 (22.1)	1	<0.001*
	**> 246**	145/366 (39.6)	1.79 (1.46–2.2)	
TnT, ng/L	**≤ 12.5**	108/496 (21.8)	1	<0.001*
	**> 12.5**	152/392 (38.8)	1.78 (1.45–2.19)	
DD, mg/mL	**≤ 0.83**	106/454 (23.3)	1	<0.001*
	**> 0.83**	160/449 (35.6)	1.53 (1.24–1.88)	
FT, mg/L	**≤ 810**	132/541 (24.4)	1	<0.001*
	**> 810**	136/387 (35.1)	1.44 (1.18–1.76)	
**ICU admission**		**n/N (%)**	**OR (95%CI)**	***p***
Total lymphocytes, cells/mm^3^	**≤ 948**	66/442 (14.9)	1.84 (1.27–2.66)	<0.001*
	**> 948**	41/505 (8.1)	1	
CD3+, cells/mm^3^	**≤ 666**	69/460 (15)	1.89 (1.3–2.74)	<0.001*
	**> 666**	38/478 (7.9)	1	
CD4+, cells/mm^3^	**≤ 359**	59/384 (15.4)	1.77 (1.24–2.54)	0.002*
	**> 359**	48/554 (8.7)	1	
CD8+, cells/mm^3^	**≤ 241**	75/507 (14.8)	1.99 (1.34–2.95)	<0.001*
	**> 241**	32/431 (7.4)	1	
B, cells/mm^3^	**≤ 77**	44/339 (13)	1.26 (0.87–1.83)	0.23
	**> 77**	54/524 (10.3)	1	
NK, cells/mm^3^	**≤ 117**	41/261 (15.7)	1.64 (1.13–2.38)	0.01*
	**> 117**	58/604 (9.6)	1	
IL-6, pg/mL	**≤ 39.5**	37/595 (6.2)	1	<0.001*
	**> 39.5**	71/364 (19.5)	3.13 (2.15–4.57)	
CRP, mg/dL	**≤ 5.3**	32/440 (7.3)	1	<0.001*
	**> 5.3**	74/513 (14.4)	1.98 (1.34–2.94)	
proBNP, pg/mL	**≤ 246**	62/528 (11.7)	1	0.28
	**> 246**	35/369 (9.5)	0.81 (0.55–1.2)	
TnT, ng/L	**≤ 12.5**	55/499 (11)	1	0.95
	**> 12.5**	43/395 (10.9)	0.99 (0.68–1.44)	
DD, mg/mL	**≤ 0.83**	52/458 (11.4)	1	0.8
	**> 0.83**	49/453 (10.8)	0.95 (0.66–1.38)	
FT, mg/L	**≤ 810**	44/548 (8)	1	<0.001*
	**> 810**	59/389 (15.2)	1.89 (1.31–2.73)	
**In-hospital death**		**n/N (%)**	**OR (95%CI)**	**p**
Total lymphocytes, cells/mm^3^	**≤ 948**	68/440 (15.5)	2.37 (1.59–3.51)	<0.001*
	**> 948**	33/505 (6.5)	1	
CD3+, cells/mm^3^	**≤ 666**	73/458 (15.9)	2.92 (1.9–4.48)	<0.001*
	**> 666**	26/476 (5.5)	1	
CD4+, cells/mm^3^	**≤ 359**	67/382 (17.5)	3.03 (2.03–4.52)	<0.001*
	**> 359**	32/552 (5.8)	1	
CD8+, cells/mm^3^	**≤ 241**	69/504 (13.7)	1.96 (1.3–2.95)	<0.001*
	**> 241**	30/430 (7)	1	
B, cells/mm^3^	**≤ 77**	56/338 (16.6)	2.7 (1.79–4.07)	<0.001*
	**> 77**	32/521 (6.1)	1	
NK, cells/mm^3^	**≤ 117**	37/259 (14.3)	1.65 (1.11–2.46)	0.01*
	**> 117**	52/602 (8.6)	1	
IL-6, pg/mL	**≤ 39.5**	38/592 (6.4)	1	<0.001*
	**> 39.5**	64/363 (17.6)	2.75 (1.88–4.02)	
CRP, mg/dL	**≤ 5.3**	29/439 (6.6)	1	<0.001*
	**> 5.3**	72/512 (14.1)	2.13 (1.41–3.22)	
proBNP, pg/mL	**≤ 246**	19/528 (3.6)	1	<0.001*
	**> 246**	74/367 (20.2)	5.62 (3.45–9.09)	
TnT, ng/L	**≤ 12.5**	12/498 (2.4)	1	<0.001*
	**> 12.5**	80/394 (20.3)	8.4 (4.65–15.15)	
DD, mg/mL	**≤ 0.83**	24/457 (5.3)	1	<0.001*
	**> 0.83**	69/452 (15.3)	2.91 (1.86–4.55)	
FT, mg/L	**≤ 810**	55/547 (10.1)	1	0.45
	**> 810**	45/388 (11.6)	1.15 (0.8–1.67)	

OR, odds ratio; 95%CI, 95% confidence interval; p, *p*-value; IL-6, interleukin 6; CRP, C reactive protein; proBNP, brain natriuretic peptide; TnT, troponin T; DD, D dimer; FT, ferritin. Significant *p*-values are highlighted in bold and marked with an asterisk (*).

Levels of IL-6 above 39.5 pg/mL are associated with higher risk of ventilatory support (OR 2.46, 95% CI: 2.01–3.01, *p* < 0.001), ICU admission (OR 3.13, 95% CI: 2.15–4.57, *p* < 0.001) and in-hospital death (OR 2.75, 95% CI: 1.88–4.02, *p* < 0.001). Patients with CRP over 5.3mg/dL are at higher risk of all analyzed outcomes: ventilatory support (OR 1.97, 95% CI: 1.58–2.46, *p* < 0.001), ICU admission (OR 1.98, 95% CI: 1.34–2.94, *p* < 0.001) and in-hospital death (OR 2.13, 95% CI: 1.41–3.22, *p* < 0.001). Other inflammatory biomarkers associated with ventilatory support are proBNP (OR 1.79, 95% CI: 1.46–2.2, *p* < 0.001), TnT (OR 1.78, 95% CI: 1.45–2.19, *p* < 0.001), DD (OR 1.53, 95% CI: 1.24–1.88, *p* < 0.001) and FT (OR 1.44, 95% CI: 1.18–1.76, *p* < 0.001). FT over mg/L is linked to higher risk of ICU admission (OR 1.89, 95% CI: 1.31–2.73, *p* < 0.001). Finally, high levels of proBNP (OR 5.62, 95% CI: 3.45–9.09, *p* < 0.001), TnT (OR 8.4, 95% CI: 4.65–15.15, *p* < 0.001) and DD (OR 2.91, 95% CI: 1.86–4.55, *p* < 0.001) are associated with in-hospital death.

### 3.4 Multivariable analysis

Multivariable analysis was performed to adjust the findings in the univariable analysis for different clinical and analytical variables that may influence the results. Binary logistic regression results are shown in Table 7 for ventilatory support, Table 8 for ICU admission and Table 9 for in-hospital death. After adjusting by confounders, CD3+ levels were independently associated with ventilatory support (aOR: 2.3, 95%CI: 1.5–3.4, p* = 0.013) and in-hospital death (aOR: 2.4, 95%CI: 1.3–4.3, p* 0.048), and CD4+ levels were an independent risk factor of in-hospital death (aOR: 2.8, 95%CI: 1.4–5.5, p* 0.045).

**TABLE 7:** Multivariate analysis adjusted by sex, age, comorbidity, SO_2_, radiological findings and laboratory biomarkers for ventilatory support.

Variable		n/N (%)	OR (95%CI)	*p*	aOR (Model 1)	p*1	aOR (Model 2)	p*2	aOR (Model 3)	p*3
Total lymphocytes, cells/mm^3^	**≤ 948**	165/438 (37.7)	1.71 (1.39–2.1)	< 0.001*	-	-	1.32 (0.88–1.96)	0.99	-	-
	**> 948**	110/499 (22)	1		-	1	-			
CD3+, cells/mm^3^	**≤ 666**	182/454 (40.1)	2.15 (1.72–2.67)	<0.001*	-	-	-	-	**2.26 (1.5**–**3.41)**	<0.013*
	**> 666**	88/471 (18.7)	1		-		-		**1**	
CD4+, cells/mm^3^	**≤ 359**	152/379 (40.1)	1.86 (1.52–2.27)	< 0.001*	1.54 (0.95–2.51)	0.99	-	-	-	-
	**> 359**	118/546 (21.6)	1		1		-		-	
CD8+, cells/mm^3^	**≤ 241**	179/500 (35.8)	1.67 (1.34–2.08)	< 0.001*	1.14 (0.72–1.8)	0.99	-	-	-	-
	**> 241**	91/425 (21.4)	1		1		-		-	
B, cells/mm^3^	**≤ 77**	126/333 (37.8)	1.8 (1.45–2.23)	< 0.001*	1.46 (0.9–2.36)	0.99	-	-	-	-
	**> 77**	109/517 (21.1)	1		1		-		-	
NK, cells/mm^3^	**≤ 117**	95/260 (36.5)	1.53 (1.24–1.9)	< 0.001*	1.37 (0.86–2.18)	0.99	-	-	-	-
	**> 117**	141/592 (23.8)	1		1		-		-	
Sex	**Female**	100/412 (24.3)	1	0.003*	1	0.99	1	0.99	1	0.99
	**Male**	177/534 (33.1)	1.37 (1.11–1.68)		1.21 (0.76–1.91)		0.96 (0.63–1.46)		1.12 (0.73–1.71)	
Age	**18**–**64**	87/433 (20.1)	1	< 0.001*	1	0.99	1	0.99	1	0.99
	**≥ 65**	190/513 (37)	1.85 (1.48–2.29)		1.04 (0.5–2.17)		1.21 (0.63–2.3)		1.16 (0.6–2.23)	
Charlson comorbidity index	**0**–**2**	78/401 (19.5)	1	< 0.001*	1	0.99	1	0.99	1	0.99
	**≥ 3**	195/537 (36.3)	1.87 (1.49–2.35)		0.93 (0.43–2.01)		0.88 (0.45–1.73)		0.96 (0.48–1.91)	
SO_2_	**≤ 94%**	86/512 (16.8)	1	<0.001*	1	0.56	1	0.04*	1	<0.013*
	**> 94%**	166/380 (43.7)	2.6 (2.08–3.26)		0.62 (0.4–0.97)		0.54 (0.36–0.81)		0.5 (0.33–0.75)	
Radiological findings	**Normal**	35/164 (21.3)	1	0.013*	1	0.99	1	0.99	1	0.99
	**Pneumonia**	238/765 (31.1)	1.46 (1.07–1.99)		0.99 (0.52–1.88)		1.15 (0.64–2.07)		1.08 (0.59–1.98)	
IL-6, pg/mL	**≤ 39.5**	110/585 (18.8)	1	< 0.001*	1	0.05	1	0.07	1	0.03*
	**> 39.5**	167/361 (46.3)	2.46 (2.01–3.01)		0.52 (0.33–0.8)		0.56 (0.38–0.84)		0.53 (0.35–0.79)	
Tocilizumab	**No**	77/637 (12.1)	1	< 0.001*	1	<0.016*	1	< 0.013*	1	<0.013*
	**Yes**	200/308 (64.9)	5.38 (4.29–6.71)		0.08 (0.05–0.12)		0.09 (0.06–0.14)		0.1 (0.06–0.15)	
CRP, mg/dL	**≤ 5.3**	83/432 (19.2)	1	< 0.001*	1	0.99	1	0.99	1	0.99
	**> 5.3**	194/512 (37.9)	1.97 (1.58–2.46)		0.9 (0.56–1.44)		0.89 (0.58–1.37)		0.93 (0.6–1.44)	
proBNP, pg/mL	**≤ 246**	116/525 (22.1)	1	< 0.001*	1	0.99	1	0.99	1	0.99
	**> 246**	145/366 (39.6)	1.79 (1.46–2.2)		0.66 (0.37–1.19)		0.77 (0.45–1.3)		0.74 (0.44–1.27)	
TnT, ng/L	**≤ 12.5**	108/496 (21.8)	1	< 0.001*	1	0.99	1	0.99	1	0.99
	**> 12.5**	152/392 (38.8)	1.78 (1.45–2.19)		0.69 (0.37–1.27)		0.67 (0.38–1.18)		0.72 (0.41–1.26)	
DD, mg/mL	**≤ 0.83**	106/454 (23.3)	1	< 0.001*	1	0.99	1	0.99	1	0.99
	**> 0.83**	160/449 (35.6)	1.53 (1.24–1.88)		0.98 (0.63–1.53)		0.96 (0.64–1.45)		0.98 (0.65–1.48)	
FT, mg/L	**≤ 810**	132/541 (24.4)	1	< 0.001*	1	0.99	1	0.99	1	0.99
	**> 810**	136/387 (35.1)	1.44 (1.18–1.76)		1.11 (0.69–1.77)		1.09 (0.71–1.68)		1.07 (0.69–1.66)	

OR, odds ratio; 95%CI, 95% confidence interval; aOR, adjusted odds ratio; p*1, adjusted *p*-value for model 1 (CD4, CD8, B, NK); p*2, adjusted *p*-value for model 2 (total lymphocytes); p*3, adjusted *p*-value for model 3 (CD3+); SO_2_, oxygen saturation; n/N, proportion; %, percentage; IL-6, interleukin 6; CRP, C reactive protein; proBNP, brain natriuretic peptide; TnT, troponin T; DD, D dimer; FT, ferritin. Significant *p*-values and confidence intervals are highlighted in bold and marked with an asterisk (*).

**TABLE 8:** Multivariate analysis adjusted by sex, age, comorbidity, SO_2_, radiological findings and laboratory biomarkers for ICU admission.

Variable		n/N (%)	OR (95%CI)	*p*	aOR (Model 1)	p*1	aOR (Model 2)	p*2	aOR (Model 3)	p*3
Total lymphocytes, cells/mm^3^	**≤ 948**	66/442 (14.9)	1.84 (1.27–2.66)	< 0.001*	-	-	1.42 (0.88–2.29)	0.99	-	-
	**> 948**	41/505 (8.1)	1		-		1		-	
CD3+, cells/mm^3^	**≤ 666**	69/460 (15)	1.89 (1.3–2.74)	< 0.001*	-	-	-	-	1.39 (0.85–2.28)	0.99
	**> 666**	38/478 (7.9)	1		-		-		1	
CD4+, cells/mm^3^	**≤ 359**	59/384 (15.4)	1.77 (1.24–2.54)	0.002*	1.32 (0.78–2.23)	0.99	-	-	-	-
	**> 359**	48/554 (8.7)	1		1		-		-	
CD8+, cells/mm^3^	**≤ 241**	75/507 (14.8)	1.99 (1.34–2.95)	< 0.001*	1.55 (0.89–2.69)	0.99	-	-	-	-
	**> 241**	32/431 (7.4)	1		1		-		-	
NK, cells/mm^3^	**≤ 117**	41/261 (15.7)	1.64 (1.13–2.38)	0.01*	1.1 (0.65–1.74)	0.99	-	-	-	-
	**> 117**	58/604 (9.6)	1		1		-		-	
Sex	**Female**	42/420 (10)	1	0.28	1	0.99	1	0.99	1	0.84
	**Male**	66/539 (12.2)	1.05 (0.74–1.51)		1.38 (0.81–2.36)		1.44 (0.87–2.38)		1.56 (0.93–2.61)	
Age	**18**–**64**	48/439 (10.9)	1	0.77	1	0.99	1	0.99	1	0.99
	**≥ 65**	60/520 (11.5)	2.10 (1.38–3.20)		0.56 (0.25–1.27)		0.57 (0.27–1.21)		0.57 (0.26–1.22)	
Charlson comorbidity index	**0**–**2**	50/405 (12.3)	1	0.36	1	0.08	1	0.08	1	0.13
	**≥ 3**	57/546 (10.4)	0.98 (0.93–1.03)		3.09 (1.36–7.07)		2.77 (1.3–5.91)		2.6 (1.21–5.58)	
SO_2_	**≤ 94%**	37/520 (7.1)	1	< 0.001*	1	0.99	1	0.99	1	0.99
	**> 94%**	61/382 (16)	2.24 (1.52–3.3)		1.23 (0.72–2.11)		0.8 (0.48–1.33)		0.79 (0.47–1.31)	
IL-6, pg/mL	**≤ 39.5**	37/595 (6.2)	1	< 0.001*	1	0.18	1	0.34	1	0.2
	**> 39.5**	71/364 (19.5)	3.13 (2.15–4.57)		0.52 (0.3–0.88)		0.58 (0.35–0.97)		0.55 (0.33–0.92)	
Tocilizumab	**No**	26/644 (4)	1	< 0.001*	1	<0.01*	1	< 0.01*	1	<0.01*
	**Yes**	82/309 (26.5)	6.58 (4.32–10)		0.13 (0.07–0.23)		0.13 (0.07–0.23)		0.13 (0.07–0.23)	
CRP, mg/dL	**≤ 5.3**	32/440 (7.3)	1	< 0.001*	1	0.99	1	0.99	1	0.99
	**> 5.3**	74/513 (14.4)	1.98 (1.34–2.94)		0.83 (0.49–1.39)		0.85 (0.52–1.4)		0.89 (0.54–1.48)	
FT, mg/L	**≤ 933**	44/548 (8)	1	< 0.001*	1	0.99	1	0.99	1	0.99
	**> 933**	59/389 (15.2)	1.89 (1.31–2.73)		0.96 (0.56–1.63)		0.88 (0.53–1.46)		0.85 (0.51–1.42)	

OR, odds ratio; 95%CI, 95% confidence interval; aOR, adjusted odds ratio; p*1, adjusted *p*-value for model 1 (CD4, CD8, B, NK); p*2, adjusted *p*-value for model 2 (total lymphocytes); p*3: adjusted *p*-value for model 3 (CD3+); SO_2_, oxygen saturation; n/N, proportion; %, percentage; IL-6, interleukin 6; CRP, C reactive protein; FT, ferritin. Significant *p*-values and confidence intervals are highlighted in bold and marked with an asterisk (*).

**TABLE 9:** Multivariate analysis adjusted by sex, age, comorbidity, SO_2_, radiological findings and laboratory biomarkers for in-hospital death.

Variable		n/N (%)	OR (95%CI)	*p*	aOR (Model 1)	p*1	aOR (Model 2)	p*2	aOR (Model 3)	p*3
Total lymphocytes, cells/mm^3^	**≤ 948**	68/440 (15.5)	2.37 (1.59–3.51)	< 0.001*	-	-	1.52 (0.87–2.63)	0.99	-	-
	**> 948**	33/505 (6.5)	1		-		1		-	
CD3+, cells/mm^3^	**≤ 666**	73/458 (15.9)	2.92 (1.9–4.48)	< 0.001*	-	-	-	-	**2.4 (1.33**–**4.33)**	0.048*
	**> 666**	26/476 (5.5)	1		–		–		**1**	
CD4+, cells/mm^3^	**≤ 359**	67/382 (17.5)	3.03 (2.03–4.52)	< 0.001*	**2.8 (1.43**–**5.48)**	0.045*	-	-	-	-
	**> 359**	32/552 (5.8)	1		**1**		-		-	
CD8+, cells/mm^3^	**≤ 241**	69/504 (13.7)	1.96 (1.3–2.95)	< 0.001*	1.35 (0.71–2.58)	0.99	-	-	-	-
	**> 241**	30/430 (7)	1		1		-		-	
B, cells/mm^3^	**≤ 77**	56/338 (16.6)	2.7 (1.79–4.07)	< 0.001*	0.77 (0.4–1.5)	0.99	-	-	-	-
	**> 77**	32/521 (6.1)	1		1		-		-	
NK, cells/mm^3^	**≤ 117**	37/259 (14.3)	1.65 (1.11–2.46)	0.013*	1.3 (0.72–2.35)	0.99	-	-	-	-
	**> 117**	52/602 (8.6)	1		1		-		-	
Sex	**Female**	42/418 (10)	1	0.58	1	0.99	1	0.99	1	0.99
	**Male**	60/537 (11.2)	1.11 (0.77–1.61)		1.07 (0.6–1.93)		1.07 (0.62–1.82)		1.1 (0.64–1.9)	
Age	**18**–**64**	7/438 (1.6)	1	< 0.001*	1	0.12	1	0.05	1	0.07
	**≥ 65**	95/517 (18.4)	11.49 (5.41–24.39)		6.23 (1.61–24.03)		6.68 (1.82–24.45)		6.16 (1.69–22.48)	
Charlson comorbidity index	**0**–**2**	7/404 (1.7)	1	< 0.001*	1	0.99	1	0.99	1	0.99
	**≥ 3**	93/543 (17.1)	9.9 (4.63–21.28)		1.4 (0.38–5.17)		1 (0.3–3.34)		1.11 (0.33–3.77)	
SO_2_	**≤ 94%**	35/518 (6.8)	1	< 0.001*	1	0.95	1	0.99	1	0.99
	**> 94%**	58/381 (15.2)	2.25 (1.51–3.36)		1.82 (0.97–3.41)		1.6 (0.9–2.84)		1.65 (0.92–2.94)	
Radiological findings	**Normal**	25/168 (14.9)	1	0.039*	1	0.68	1	0.22	1	0.36
	**Pneumonia**	73/768 (9.5)	0.68 (0.42–0.97)		2.21 (1.02–4.81)		2.29 (1.15–4.57)		2.23 (1.08–4.59)	
IL-6, pg/mL	**≤ 39.5**	38/592 (6.4)	1	< 0.001*	1	0.96	1	0.99	1	0.99
	**> 39.5**	64/363 (17.6)	2.75 (1.88–4.02)		0.57 (0.32–1.03)		0.7 (0.41–1.19)		0.66 (0.38–1.15)	
Tocilizumab	**No**	48/643 (7.5)	1	< 0.001*	1	0.03*	1	0.1	1	0.13
	**Yes**	54/308 (17.5)	2.35 (1.63–3.38)		0.38 (0.21–0.7)		0.47 (0.27–0.82)		0.48 (0.27–0.85)	
CRP, mg/dL	**≤ 5.3**	29/439 (6.6)	1	0.01*	1	0.99	1	0.99	1	0.99
	**> 5.3**	72/512 (14.1)	2.13 (1.41–3.22)		0.78 (0.39–1.55)		0.7 (0.38–1.3)		0.67 (0.36–1.27)	
proBNP, pg/mL	**≤ 246**	19/528 (3.6)	1	< 0.001*	1	0.65	1	0.99	1	0.99
	**> 246**	74/367 (20.2)	5.62 (3.45–9.09)		0.45 (0.21–0.98)		0.64 (0.32–1.26)		0.55 (0.27–1.12)	
TnT, ng/L	**≤ 12.5**	12/498 (2.4)	1	< 0.001*	1	0.99	1	0.59	1	0.61
	**> 12.5**	80/394 (20.3)	8.4 (4.65–15.15)		0.46 (0.19–1.11)		0.45 (0.2–1)		0.45 (0.2–1.01)	
DD, mg/mL	**≤ 0.83**	24/457 (5.3)	1	< 0.001*	1	0.65	1	0.41	1	0.52
	**> 0.83**	69/452 (15.3)	2.91 (1.86–4.55)		0.53 (0.28–0.98)		0.54 (0.31–0.95)		0.55 (0.3–0.98)	

OR, odds ratio; 95%CI, 95% confidence interval; aOR, adjusted odds ratio; p*1, adjusted *p*-value for model 1 (CD4, CD8, B, NK); p*2, adjusted *p*-value for model 2 (total lymphocytes); p*3, adjusted *p*-value for model 3 (CD3+); SO_2_, oxygen saturation; n/N, proportion; %, percentage; IL-6, interleukin 6; CRP, C reactive protein; proBNP, brain natriuretic peptide; TnT, troponin T; DD, D dimer. Significant *p*-values and confidence intervals are highlighted in bold and marked with an asterisk (*).

Tocilizumab was a protective factor for ventilatory support (model 1: aOR 0.08, 95%CI: 0.05–0.12, p* = 0.016; model 2: aOR 0.09, 95%CI: 0.06–0.14, p* = 0.013; model 3: aOR 0.1, 95%CI: 0.06–0.15, p* = 0.013) and ICU admission (model 1: aOR 0.13, 95%CI: 0.07–0.23, p* = 0.01; model 2: aOR 0.13, 95%CI: 0.07–0.23, p* = 0.01; model 3: aOR 0.13, 95%CI: 0.07–0.23, p* = 0.01), but this effect on in-hospital death was only statistically significant in model 1 (aOR 0.38, 95%CI: 0.21–0.7, p* = 0.03). Low oxygen saturation (under 94%) was an independent risk factor of ventilatory support in model 2 (aOR 1.85, 95%CI: 1.23–2.78, p* = 0.04) and model 3 (aOR 2, 95%CI: 1.33–3.03, p* = 0.013).

There are also some clinical variables with a non-significant p value after Bonferroni correction, but with an effect size and confidence interval that suggest an association with poor outcomes that are worth pointing out. On the one hand, high CCI shows a potential association with ICU admission (model 1: aOR 3.09, 95%CI: 1.36–7.07, p*0.08; model 2: aOR 2.77, 95%CI: 1.3–5.91, p*0.08; model 3: aOR 2.6, 95%CI: 1.21–5.58, p*0.13). On the other hand, in-hospital death tends to be higher in patients aged 65 or older (model 1: aOR 6.23, 95%CI: 1.61–24.03, p*0.12; model 2: aOR 6.68, 95%CI: 1.82–24.45, p*0.05; model 3: aOR 6.16, 95%CI: 1.69–22.48, p*0.07) and with pneumonia on admission (model 1: aOR 2.21, 95%CI: 1.02–4.81, p*0.68; model 2: aOR 2.29, 95%CI: 1.15–4.57, p*0.22; model 3: aOR 2.23, 95%CI: 1.08–4.59, p*0.36).

All multivariable logistic regression models showed a significant improvement over the null model, as assessed by the likelihood ratio chi-square test, with *p* < 0.001 for all models in the three outcomes tested: ventilatory support, ICU admission and in-hospital death. Omnibus chi square statistics and *p*-values, -2 log likelihood values and *p*-values for Hosmer and Lemeshow’s test for each multivariate model are shown in Supplementary Table 1. Since the models evaluate different outcomes, these values should be interpreted as descriptors of goodness of fit rather than for direct comparison between models.

## 4 Discussion

The findings of this study show that COVID-19 patients with T CD3+ cells on admission below 666 cells/mm^3^ have higher risk of ventilatory support and in-hospital death, and T CD4+ cells levels below 359 cells/mm^3^ are associated with higher risk of in-hospital death. These findings are consistent with the currently available literature. The first meta-analysis including studies made in China and Korea during the first moments of the COVID-19 pandemic showed a significant decrease on lymphocyte counts (12, 13). Huang et al. (12) found that the absolute cell counts decreased in the severe/critical group for all subsets, although the decrease in CD4+ and CD8+ cells was larger than B and NK cells. Two of the included studies performed a multivariate analysis: Du et al. (14), in which CD8+ cells below 75 cells/mm^3^ were associated with in-hospital death; and Chen et al. (15), with an inverse, independent association between CD4+ on admission and ICU admission. In the meta-analysis of Yan et al. (13), the levels of CD3+, CD4+, CD8+ and B cells were lower in patients with severe disease compared to mild and critical disease compared to mild.

More observational studies have shown that T cells (CD3+, CD4+ and CD8+) are associated with poor outcomes in COVID-19 infection, with some differences between T cell subsets and outcomes. Chu et al. (16) found that patients with aggravated disease showed lower counts of CD3+ and CD4+ T cells, while in Wen et al.’s cohort (17), low CD8+ levels were associated with disease severity, but only CD4+ were independently associated with in-hospital death.

These results are consistent with other cohort studies performed in similar populations as ours. Calvet et al. (18) measured lymphocyte populations in 30 hospitalized patients in a tertiary care hospital in Spain, and although older patients were excluded, the results suggested a correlation between lymphocyte subsets and critical disease, especially for CD4. Cantenys-Molina et al. (19) gathered a bigger Spanish cohort of 701 hospitalized patients and found that patients with in-hospital death had lower levels of CD3, CD4, CD8 and B cells, and higher NK cells. In multivariate analysis, the independent risk factors for in-hospital death were age, CD4 ≤ 500 cells/μl, CD8 ≤ 100 cells/μL and NK ≥ 30% (19). Lastly, the cohort study from Iannetta et al. (20) included 160 patients admitted in an Italian hospital, analyzing in-hospital death and ventilatory support. The results were similar to our study; total CD3+ cells and CD4+ cells were independently associated with in-hospital death (20). Iannetta et al. also calculated the ideal cutoffs within the cohort associated with in-hospital death, which were 524 cells/mm^3^, for CD3+, and 369 cells/mm^3^, for CD4+ (20). In the case of NK cells, some studies show an increase in patients with severe disease (19), while others show a consistent decrease (21). In our study, NK cells tend to be lower in patients with severe outcomes; however, this trend loses statistical significance when adjusted for another clinical and analytical variables. This could be explained by the functional heterogeneity of NK cells, with different subsets, some with more cytotoxicity (CD56dim+), while others are less cytotoxic, but show a high capacity of cytokine synthesis (21). In our study, the cutoff with higher Youden index was 666 cells/mm^3^, in the case of CD3+, higher than the cutoff of Iannetta et al. (20) and 359 cells/mm^3^, for CD4+, very similar to Iannetta et al. (20), and lower than the 500 cells/mm^3^ used by Cantenys-Molina et al. (19), which is the cutoff between category 1 and 2 in the Atlanta human immunodeficiency virus classification (22). In our cohort, the CD4+ cutoff point of 500 cells/mm^3^ had a sensitivity of 85.9% and a specificity of 41.1%, whereas the cutoff point of 359 cells/mm^3^ had a sensitivity and specificity of 67.7% and 63.3%, respectively. This shows that setting the cutoff point lower than 500 cells/mm^3^ rises specificity at the cost of sensitivity. Therefore, the cutoff point of 353 (350) cells/mm^3^ may be used to complement the cutoff of 500 cells/mm^3^ depending on the clinical context, with one being the screening cutoff and the second the one the more specific for in-hospital death.

The findings in our study suggest that the impairment of T cell response is associated with poor outcomes in COVID-19 infection, leaning more to the role of CD4+ when it comes to in-hospital death. T cell exhaustion is a well-known phenomenon in severe COVID-19, which consists of not only a reduction in the number and proliferation of T cells, but also of an impairment of its effector functions through metabolic and epigenetic dysregulation and transcriptional reprogramming, an increase in the expression of inhibitor receptors, such as cytotoxic T-lymphocyte-associated protein 4 (CTLA-4), programmed cell death protein-1 (PD-1) and T-cell immunoglobulin and mucin-domain containing-3 (TIM-3), and a decrease in cytokine production (23). The key factors behind T cell exhaustion are prolonged antigenic stimulation and inflammatory signals (23).

In this sense, the cytokine storm plays a crucial role in T-cell depletion and exhaustion; cross-sectional studies in COVID-19 patients have shown an inverse correlation between levels of T cells and concentrations of cytokines well known as participants in the inflammatory dysregulation in COVID-19 (24, 25), such as interferon gamma (24), IL-1beta (24), IL-10 (25), tumor necrosis factor alpha and IL-6 (24, 25). The likely mechanism behind this correlation is gasdermin E-mediated pyroptosis, mainly induced by IL-6 (24).

It is worth mentioning the relevance of IL-6 in the inflammatory response to COVID-19. IL-6 is a glycoprotein produced by both immune (T cells, B cells, macrophages, mast cells and dendritic cells) and stromal cells (keratinocytes, fibroblasts, mesangial cells and vascular endothelial cells), mainly activated by tumor necrosis factor and IL-1beta (26). IL-6 can induce signal transduction through two different pathways. In the first, known as classic (26) or cis-activating pathway (27), IL-6 binds to its membrane bound receptor (mbIL-6R) and activates a signaling pathway depending on JAK kinases (26). Cis-signaling has an immunomodulator, protective role, controlling different metabolic pathways (27). In the second pathway, known as trans-signaling, IL-6 binds to soluble IL-6 receptor (sIL-6R), generated by cleavage of mbIL6-R by metalloprotease ADAM17 (26, 27), and it is linked to inflammation, macrophage differentiation, immune cell recruitment and impairment of T regulator cells (27).

Due to this role in systemic inflammation and its interaction with T cells, elevated levels of IL-6 have been associated in the literature to severe COVID-19, and targeted therapies against IL-6, such as tocilizumab, have been associated with better outcomes of COVID-19 (27). In our study, IL-6 is the inflammatory biomarker with a more consistent association with ventilatory support, ICU admission and in-hospital death. In the multivariable analysis, however, this effect disappears, likely due to the use of tocilizumab in severely ill patients, which may be a confounder.

Reprising the role of T cells in COVID-19, CD4+ cells play a central role in the immune adaptive response to viral infections through diverse pathways (5), which may explain why the low levels of CD4+ cells are independently associated with in-hospital death in this cohort. First, the activation of T helper 1 cells through the major histocompatibility complex II in antigen presenting cells stimulates the production of interferon gamma, an antiviral cytokine (28). CD4+ cells also promote the stimulation and differentiation of other lymphocyte subsets, such as CD8+ T cells and B cells, which in turn enhances cytotoxic and humoral immune adaptive response (5).

Moreover, CD4+ cells also intervene in two driver phenomena of the immune response: cross-presentation and bystander effect. The bystander effect consists of the activation of CD4+ cells directly by inflammatory cytokines (IL-6, IL-2 and type I interferons), and thus, independent of antigen-T cells receptor (TCR) interaction (29). This mechanism, which integrates both innate and adaptive immunity, can improve immune response to viral infections, but in the specific case of COVID-19, the excess of molecules involved in the cytokine storm, especially IL-6, may exacerbate inflammation and tissue damage (27).

Cross presentation is the process by which exogenous antigens are internalized by dendritic cells and presented on major histocompatibility complex (MHC) class I (MCH-I) molecules directly to CD8+ cells, which is especially important in the adaptive immune response against viruses and peripheral immune tolerance (30). Although mainly involving CD8+ cells, cross presentation also involves CD4+ cells; on the one hand, cross-reactive heterologous CD4+ can be preactivated by inflammatory cytokines (tumor necrosis factor alpha, IL-6 and IL-10), in clinical scenarios of acute infections and inflammation (31). On the other hand, cytotoxic T follicular helper cells, a subset of CD4+ cells, can express chemokines that recruit dendritic cells to the places where the immune response is taking place, promoting cross-presentation to CD8+ cells (32).

This study has several strengths. It includes a large cohort (*n* = 959) of consecutively enrolled patients from a tertiary hospital, with early (within 24 h of admission) determination of lymphocyte subpopulations by standardized immunophenotyping. The clinical outcomes assessed— non-invasive ventilatory support, ICU admission, and in-hospital death—are robust, clinically relevant and easy to reciprocate in other studies. Multivariable analysis included clinical characteristics, treatments and inflammatory biomarkers, which enhances the precision of the estimates and reduces potential confounding.

However, this study also has limitations. Its retrospective, single-center, pre-vaccination design limits generalization and may imply potential residual confounding from unmeasured variables, such as exact baseline severity or duration of illness before admission. Immunophenotyping was not available for all hospitalized patients, which entails possibility of selection bias. Furthermore, some specific lymphocyte populations, such as T regulatory cells (CD3+ CD4+ CD25+bright/CD127+dim) and Th17 cells (CCR6+) were not included in the immune phenotyping. Since lymphocyte populations were measured only once on admission, dynamic analysis during hospitalization was not possible. Grouping the comorbidities in the CCI, while useful to simplify the statistical analysis and to avoid multiple comparisons, may mask the role of individual diseases with an established role in the immunopathogenesis and severity of COVID-19, such as lung diseases, autoimmune conditions and chronic illnesses with low-grade inflammation. The cutoff values were derived from the same dataset using the Youden index, lacking external validation; moreover, they may overestimate the effect compared to the continuous analysis. When correcting type I error, Bonferroni correction is a simple and effective, but also conservative approach with an increased risk of false negatives, which should be considered when interpreting the results. Lastly, COVID-19 treatment changed substantially during the study period, which could introduce uncontrolled variability in outcomes.

The clinical application of the findings of this study depends on the availability of flow cytometry, which, while broadly available in our setting (Europe), may be of limited access in other countries. In these cases, though it is true that T cells, especially CD4+, are the lymphocytes with the strongest association to poor outcomes in COVID-19, the absolute lymphocyte count can be useful when stratifying patients, especially in low-resource settings. In any case, though the use of cutoffs can help to quickly identify risk groups in a daily clinical setting, they should be a complement, never a substitute, to the clinical assessment of the patient.

As COVID-19 cases, though still relevant, have greatly diminished, future research perspectives could be focused on extrapolating the findings obtained during the pandemic to other viral infections. There is evidence that suggests a possible relationship between changes in lymphocyte counts and clinical prognosis of different viral infections, such as influenza A (33) and B (34), respiratory syncytial virus (35), Epstein-Barr virus (36), cytomegalovirus (37) and herpes simplex virus (38). However, these studies are either old (38), performed on pediatric populations (33–35) or with healthy controls (35, 36), which drives the need for more prospective studies on hospitalized populations with similar methods as the ones used in COVID-19.

## 5 Conclusion

In conclusion, patients with CD3+ T-cell counts below 666 cells/mm^3^ showed am increased risk of ventilatory support (aOR: 2.3, 95%CI: 1.5–3.4, *p* < 0.013) and in-hospital death (aOR: 2.4, 95%CI: 1.3–4.3, *p* 0.048); and patients with CD4+ T-cell levels below 353 cells/mm^3^ have an increased risk of in-hospital death (aOR: 2.8, 95%CI: 1.4–5.5, *p* 0.045), compared with patients above the threshold, independently of patient characteristics, treatments received, or inflammatory biomarker levels. These results support the role of adaptive immunity in SARS-CoV-2 infection. It would be interesting to perform similar perspective cohort studies on hospitalized patients with other viral infections, to comprehend the relationship between immune response and outcome, as well as to identify high-risk patients in a clinical setting.

## Data Availability

The raw data supporting the conclusions of this article will be made available by the authors, without undue reservation.
