# Supplementary material for: Low CD3+ and CD4+ T cell levels predict need for ventilatory support and in-hospital mortality in patients with COVID-19: a retrospective cohort study
Source: Front Med (Lausanne). 2026 Jan 26;13:1740358. doi: 10.3389/fmed.2026.1740358 (PMC12883402; doi:10.3389/fmed.2026.1740358)
Supplement: Supplementary file 1 [file Table_1.docx]

**Supplementary Material**

**Low CD3+ and CD4+ T-cell levels predict need for ventilatory support and in-hospital mortality in patients with COVID-19: a retrospective cohort study**

Ester Lobato-Martínez^1*^, Óscar Moreno-Pérez^2,3^, Silvia Otero-Rodríguez^4^, Raquel García-Sevila^3,5^, Francisco Marco-de-la-Calle^6^, Rosario Sánchez-Martínez^1,3^, Esperanza Merino-de-Lucas^3,4^, José-Manuel Ramos-Rincón^1,3^

^1^ Department of Internal Medicine, Dr. Balmis General University Hospital, Alicante Institute for Health and Biomedical Research (ISABIAL), Alicante, Spain.

^2^ Department of Endocrinology and Nutrition, Dr. Balmis General University Hospital, Alicante Institute for Health and Biomedical Research (ISABIAL), Alicante, Spain.

^3^ Department of Clinical Medicine, Miguel Hernández University, Alicante, Spain.

4 Infectious Diseases Unit, Dr. Balmis General University Hospital, Alicante Institute for Health and Biomedical Research (ISABIAL), Alicante, Spain.

^5^ Department of Pneumology, Dr. Balmis General University Hospital, Alicante Institute for Health and Biomedical Research (ISABIAL), Alicante, Spain.

^6^ Department of Immunology, Dr. Balmis General University Hospital, Alicante Institute for Health and Biomedical Research (ISABIAL), Alicante, Spain.

**Supplementary Figure 1**. Gating strategy and hierarchy for lymphocyte populations.


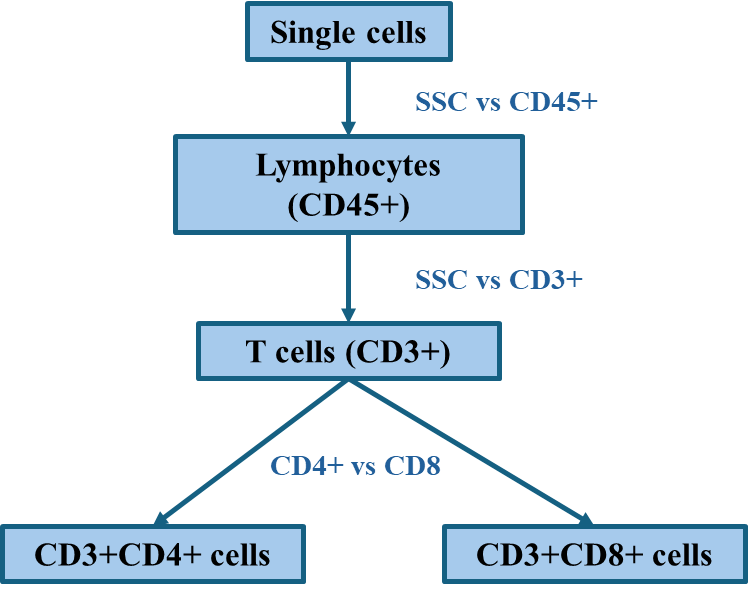

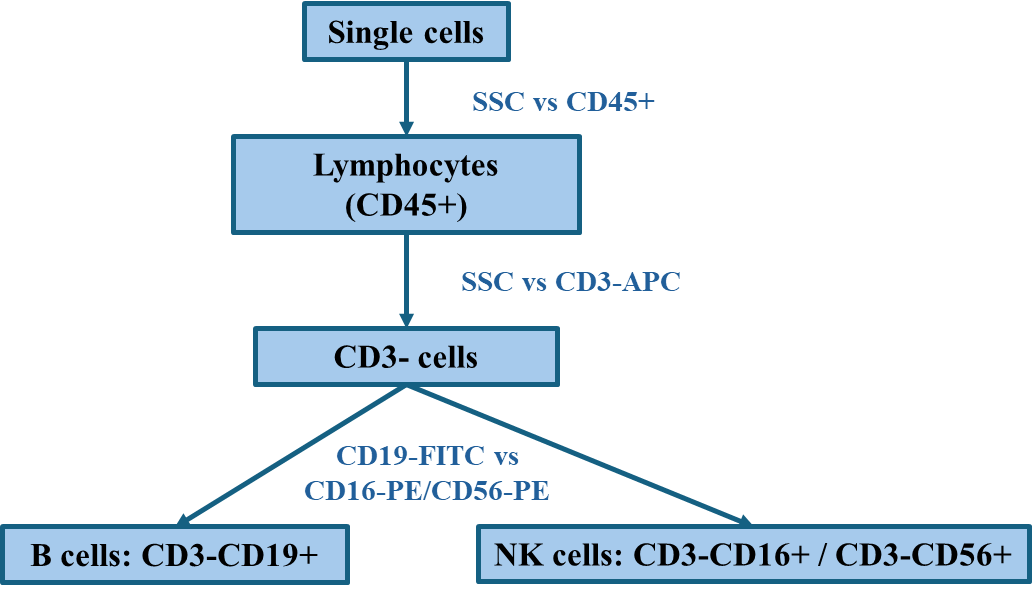


CD: cluster of differentiation; SSC: side scatter

**Supplementary Table 1.** Goodness of fit of multivariable models.

| **Outcome** | | **Ventilatory support** | **ICU admission** | **In-hospital death** |
| --- | --- | --- | --- | --- |
| **Model 1: CD4+, CD8+, B, NK** | **-2 log likelihood** | 557.47 | 432.34 | 327.89 |
|  | **Omnibus statistic** | 270.09 | 115.58 | 131.79 |
|  | **Chi square p** | **<0.001*** | **<0.001*** | **<0.001** |
|  | **Hosmer-Lemeshow p** | **0.48** | 0.017 | **0.87** |
| **Model 2: total lymphocytes** | **-2 log likelihood** | 657.13 | 478.42 | 391.67 |
|  | **Omnibus statistic** | 275.41 | 116.92 | 123.43 |
|  | **Chi square p** | **<0.001** | **<0.001** | **<0.001** |
|  | **Hosmer-Lemeshow p** | **0.09** | **0.13** | **0.41** |
| **Model 3: CD3+** | **-2 log likelihood** | 638.63 | 473.38 | 378.27 |
|  | **Chi-square statistic** | 275.27 | 114.3 | 125.28 |
|  | **Chi square p** | **<0.001*** | **<0.001** | **<0.001** |
|  | **Hosmer-Lemeshow p** | **0,77** | **0.32** | **0.6** |

p values that indicate adequate goodness of fit of the model are highlighted in **bold**
